# Precision nutrition across climates: decoding diet, tradition, and genomic adaptations from South Asia to the Arctic

**DOI:** 10.3389/fnut.2025.1638843

**Published:** 2025-08-14

**Authors:** Ajai Kumar Pathak, Anna Kolesnikova, Deepika Uttam Sambreker, Elin Org, Toomas Kivisild

**Affiliations:** ^1^Estonian Biocentre, Institute of Genomics, University of Tartu, Tartu, Estonia; ^2^Department of Human Genetics, KU Leuven, Leuven, Belgium; ^3^Institute of Computer Science, University of Tartu, Tartu, Estonia; ^4^Estonian Genome Center, Institute of Genomics, University of Tartu, Tartu, Estonia

**Keywords:** genetic variation, natural selection, traditional diet, South Asia, Arctic, nutrigenetics, precision nutrition, genome nutrition

## Abstract

Human populations have developed distinct genetic adaptations to diet in response to changes in lifestyle and environments in which they live. Particularly contrasting patterns of dietary adaptations are expected in populations living in tropical versus extreme cold environments. This article explores the genetic, dietary, and microbiome-related adaptations in populations of South Asia and the Arctic. We review adaptations related to high-carbohydrate, plant-based diets in South Asians and compare these against adaptations in Arctic populations who have evolved to rely on fat- and protein-rich diets from marine and animal sources. We discuss how these genetic adaptations interact with traditional diets and microbiomes, and the implications for chronic disease risks as modern, westernized diets disrupt ancestral gene-diet-microbiome interactions. By comparing these regions, we highlight the need for genome-based nutrition created strategies that account for genetic diversity, local dietary traditions, and environmental context to promote precision health and prevent diet-related chronic diseases. This analysis offers new insights into how nutrition, culture, and genetics intersect in shaping population-specific health outcomes.

## 1 Introduction

**Figure 1:**
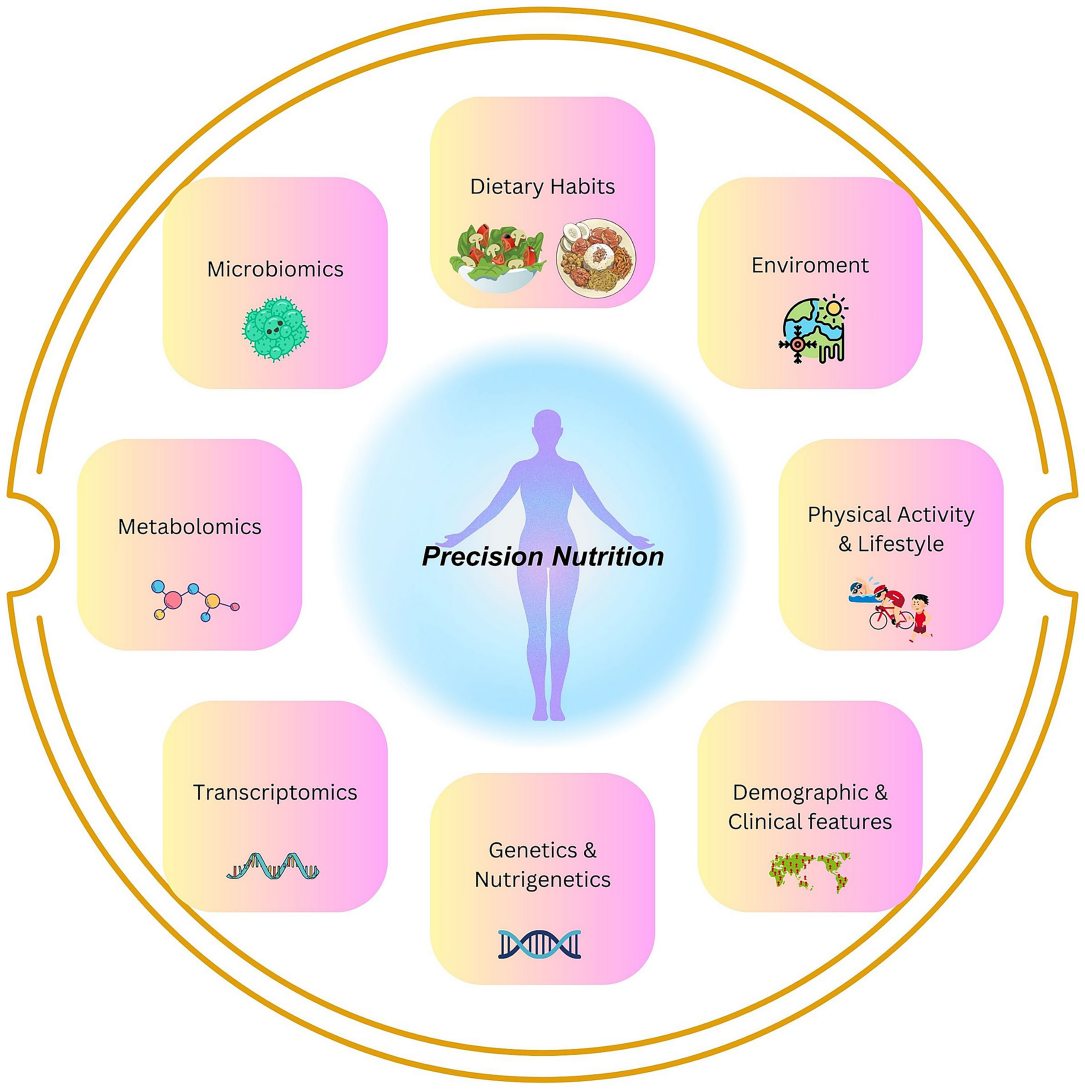
Precision Nutrition: key areas important for the understanding of inter-individual dietary differences and their impact on health covered in this review.

The advancing field of genomics and integration of multi-omics have spurred the field of genomic nutrition, leading to the era of precision nutrition (PN). It has become possible to tailor dietary recommendations based on individual differences in genes, microbiomes, metabolic responses, environments, and lifestyles (Figure 1). The approach of PN evolved due to the fact that nutritional needs and responses can vary significantly from person to person, thus contesting the traditional “one-size-fits-all” dietary guidelines (1, 2). The PN integrates multi-omics (genomics, proteomics, metabolomics, and microbiomics), data analytics, and artificial intelligence, seeking to provide more effective strategies for reducing the health burdens and preventing chronic diseases (3, 4). Concisely, the PN focuses on understanding how a particular diet and its nutrients affect individuals differently by analyzing how genetic variations can influence individual responses to dietary components (e.g., fats or carbohydrates), thereby optimizing dietary interventions for improved health outcomes (5). Thus, understanding the genetic variations, shaped by adaptation to diet which is broadly influenced by tradition/religion for generations or environmental factors (2, 6) (e.g., tropical vs. cold temperature), may be beneficial to the PN approach.

The human dietary landscape has undergone significant transformations throughout the evolutionary history of our species (2, 6, 7) shaped not only by regional environmental factors but also by deep-rooted cultural and religious traditions. Among these, three stand out as turning points. Firstly, the shift from non-cooked to cooked food led by the discovery and control of fire (8) that facilitates the breakdown of complex carbohydrates and proteins to easily digestible substances improving the nutrients and energy gains from meat and plant-based foods of early humans (9). Subsequent shift marks the momentous transition from hunter-gatherer to agricultural and pastoral subsistence at the onset of the Neolithic period (10); however, recent studies suggest these transitions as continued processes spanning millennia and across multiple source regions (11–13). The final and the relatively recent, profound shift to modern “industrialized” diet characterized intensively by farming and ultra-processed foods (14), triggered by the urbanization and Industrial Revolution. Such transitions shaped the adaptive patterns of genes, e.g., shift to starch-rich plant-based diets increased selection for salivary amylase gene copy number, while the advent of dairying (~5–7 kya) fueled the evolution of lactase persistence alleles in pastoral societies (2, 6, 7).

Moreover, the religious and traditional practices—from Islamic halal requirements and Jewish kosher laws to Hindu vegetarianism and Buddhist dietary principles—have relatively recently started to further diversify dietary patterns across human societies (15, 16). These ‘ancient wisdom’-based dietary guidelines, often entwined with seasonal variations and local ecological knowledge have shaped not only food choices but also meal timings (as in Ramadan fasting) and food preparations. For example, religious groups that abstain from alcohol, such as Muslims and certain Hindu communities, may influence the selection pressures on genes related to alcohol metabolism. The frequency of the derived class I alcohol dehydrogenase (*ADH*) variant, *ADH1BArg47His* (*rs1229984*)—linked to alcohol tolerance, is lower in India compared to western and eastern Asia (17, 18). This variant is associated with increased enzyme activity, leading to faster alcohol breakdown and potentially reduced risk of alcoholism. Hence, the selection pressure in communities where alcoholism is not abstained might have driven this variant to higher frequency. Conversely, the reduced selective pressure on alcohol tolerance in population groups where religious practices prescribe abstinence may have reduced its frequency (18, 19). Another example of ancient wisdom-based diet is traditional vegetarianism practiced predominantly in South Asia. In South Asia, religious and philosophical traditions like Hinduism, Buddhism, and Jainism promote vegetarianism or restricted consumption of animal-derived foods. This dietary pattern is supported by genetic adaptations. Populations in South Asia have a high frequency of the “vegetarian allele (*rs66698963*)”—an insertion in the second gene of the fatty acid desaturase (*FADS*) cluster, *FADS2* gene. This genetic variant enhances the conversion of short-chain omega-3 and omega-6 fatty acids into their longer-chain derivatives eicosapentaenoic acid (EPA) and docosahexaenoic acid (DHA) from plant-based foods. This genetic variant provides nutritional benefits in predominantly vegetarian diets historically shaped by religious and ethical beliefs (20, 21). Such dietary practices have potentially contributed to population-specific genetic adaptations and distinct metabolic responses that affect the health outcomes (1, 2).

The modern departure from traditional dietary practices (briefly discussed at the end of this section) and the rapid adoption of ultra-processed foods, has created an unparalleled nutritional environment to which our genetic architecture may not be optimally adapted (22, 23). This mismatch potentially contributes to the rising prevalence of diet-related disorders, with varying impacts across different populations (24, 25).

The study of diet-related genetic adaptations, therefore, provides unique insights into both our species’ nutritional history and the genetic architecture underlying concurrent population differences in dietary responses and metabolism. This intersection of dietary patterns, traditional practices, and genetic adaptation not only illuminates our past but also holds crucial implications for understanding modern population-specific nutritional requirements, metabolic responses, and health outcomes that have implications over the budding area of precision nutrition. Such insights are particularly valuable in our current era, where traditional dietary wisdom meets modern nutritional science in the quest for optimal human health (6, 26).

South Asia with the geographical border ranging from India, Pakistan, Bangladesh, Nepal, Sri Lanka, Maldives, Bhutan to Afghanistan (27) hosts around one fourth of the current world population. Contemporary South Asians exhibit high diversity not only in culture, morphology and language but also harbor a uniquely higher genetic diversities among continental populations except for Africa (28–30). Such a distinct diversity is also seen in climate, varying from tropical in the south to the temperate in the northern regions of the Indian subcontinent, and in landscape—ranging from high mountains to low-lying regions (31). Markedly diverse ethnic groups of India are generally divided into four language families; majority speaks Indo-European and Dravidian, while the remaining people are Austroasiatic and Sino-Tibetan speakers (32). However, the well-structured peopling of India is broadly layered into three categories: the ‘tribes’—constituting around 8% of Indian populations; the ‘castes’ constituting the majority of the Indian people; and other groups—comprising the remaining minorities with different religious beliefs and histories (33–35). Most of the caste populations follow pastoral and farming subsistence practices, while tribal groups are generally characterized by traditional subsistence modes such as hunting-gathering and foraging. Both the caste and tribal populations (especially Indo-European and Dravidian speaking) groups broadly practice Hindu religion and patrilineal endogamy. The genetic makeup of South Asians is broadly comprised of two main ancestry components: an indigenous South Asian component that is distantly related to Andamanese hunter-gatherers, and a West Eurasian component related to ancient and modern Europeans and West Asians (36–38). A recent study has further clarified the West Asian patrilineal contribution to South Asians (39). Whereas the Austroasiatic and Sino-Tibetan speakers also harbor Southeast and East Asian related ancestry components (36, 40).

Arctic and subarctic regions are inhabited by diverse indigenous populations who have adapted to some of the planet’s most extreme environments. Prominent among these groups are the Inuit, residing across Greenland, northern Canada, and Alaska; the Sámi, inhabiting northern parts of Norway, Sweden, Finland, and Russia’s Kola Peninsula; and various indigenous peoples of Siberia, such as the Chukchi, Evenks, and Nenets (41, 42). These communities have traditionally relied on subsistence activities like hunting, fishing, and reindeer herding, practices finely tuned to their specific environments and are facing now new adaptation challenges due to the climate change (43).

For instance, the Inuit have developed sophisticated hunting techniques for marine mammals, while the Sámi are renowned for their reindeer herding (44). Despite harsh climates, these populations have rich cultural traditions, languages, and social structures reflecting a deep connection to their lands. However, challenges include climate change, resource exploitation, and cultural assimilation, threatening traditional ways of life and heritage (45). The Yakuts, a nomadic people from the southern regions, migrated to northeastern Siberia between the 11th and 13th centuries AD (46). However, the Russian expansion into Siberia in the 17th century, forced the Yakutians to relocate and settle in Central Yakutia that led to transformation of their lifestyle and diet (46, 47). The dietary practices among the Yakuts vary across different regions. While the general population adopted a diverse diet, including a wide variety of herbivores, fish, and other foods, the elite group maintained a more stable diet based on meat and milk (48).

The genetic make-up of contemporary Arctic populations, particularly the indigenous peoples such as Inuit and Yupik, and Na-Dene and Eskimo-Aleut speakers predominantly comprises the significant genetic contribution from Siberian ancestry (East Asian lineage), plausibly inherited through the early settlers of the Arctic, the Flegontov et al. (49) and Waples et al. (50). However, these population have undergone through extensive admixture and adaptation, leaving the imprint on the genetic composition of some of the Inuit populations who harbor around one fourth of their genetic ancestry similar to that of European populations (50–52). Native Siberians populations are genetically structured and exhibit genetic differentiation between regions inside Siberia at levels comparable to continental groups. Four distinct ancestry profiles are characteristic to populations of Northeastern, Central, South and West Siberia, each with different proportions of European/East Asian and indigenous components (53). The Southern Siberian ancestry profile is prevalent in Altaian-Kizhi, Teleuts, Shors, and Buryats, Central Siberian ancestry is characteristic to Yakuts, Evenks, and Evens, while the Northeastern Siberian ancestry is most pronounced in Chukchi, Eskimo, and Koryaks, being akin to North American and Greenland Inuit ancestry. Ancient DNA studies (54, 55) have supported three migratory events that have shaped the genetic ancestry of Siberians.

The initial peopling by Paleolithic Ancient North Siberians, distantly related to West Eurasian hunter-gatherers, was followed by the East Asian-related Ancient Paleo-Siberians, prevalent in present-day far-northeast Siberians and Native Americans. Finally, a Holocene migratory event of Neo-Siberians, related to other people of East Asia, which is predominantly found in many contemporary Siberians.

**Figure 2:**
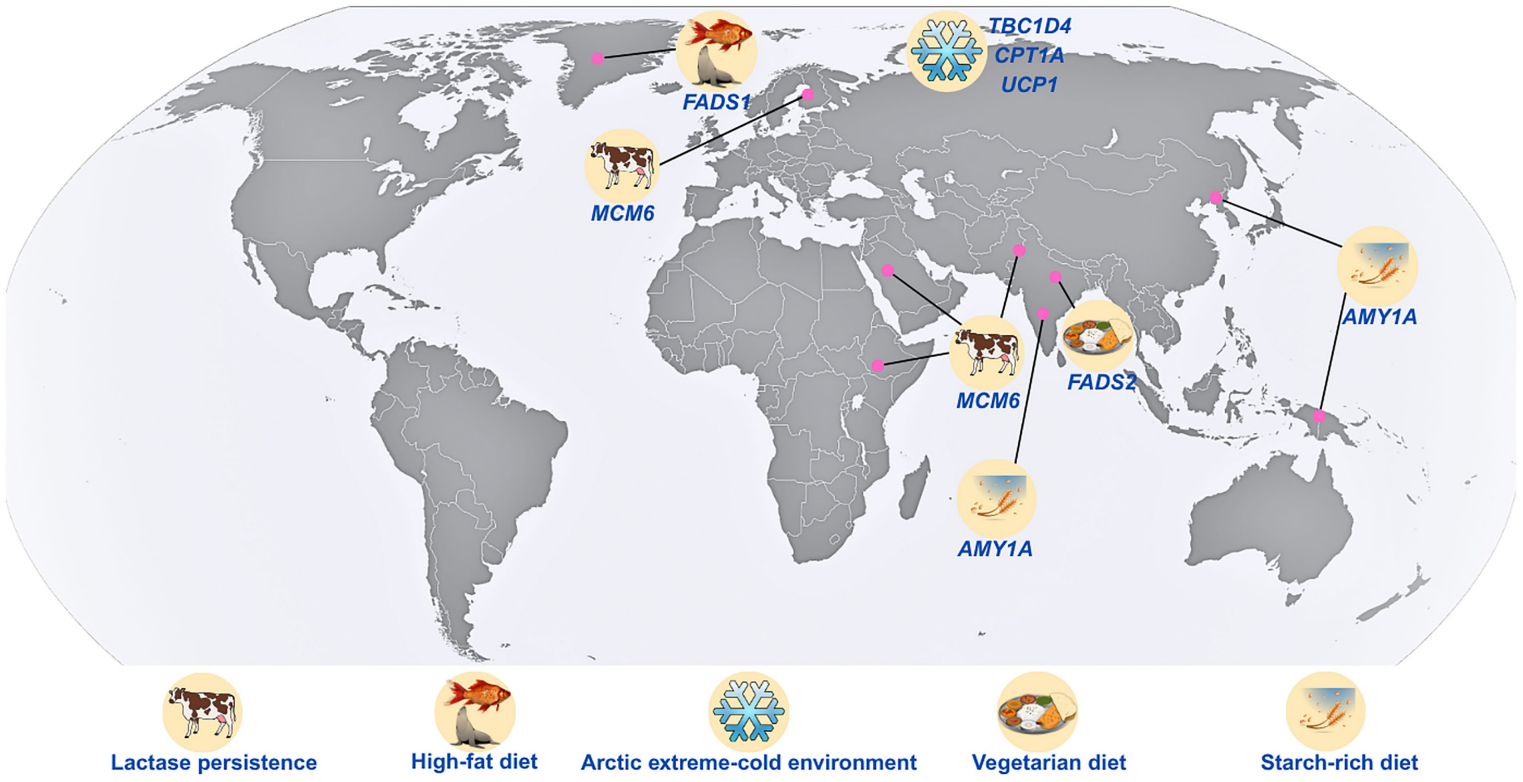
Different dietary, genetic, and environmental features that distinctly characterize the Arctic and South Asian populations. *AMY1A*, amylase alpha 1A gene; *LCT*, lactase gene; *MCM6*, minichromosome maintenance complex component 6; *FADS1*, fatty acid desaturase 1; *FADS2*, fatty acid desaturase 2; *CPT1A*, carnitine palmitoyl transferase 1A; *TBC1D4*, *TBC1* domain family member 4; and *UCP1*, uncoupling protein 1.

The environmental and dietary contrasts between South Asia and the Arctic exemplify the effects of selective pressures on dietary adaptation (Figure 2). While the tropical, agrarian climate of South Asia has historically promoted high-carbohydrate, plant-based diets, complemented by spices and fermented foods (56). Around 35% of the Indian population follows the traditional lacto-vegetarian diet practice for generations (57, 58). Arctic populations have evolved adaptations to high-fat, animal-based diets, with minimal plant consumption, to meet the energy demands of a frigid environment (51). These vastly different nutritional ecologies imposed markedly distinct selective pressures on metabolic genes.

This article will compare South Asia and Arctic populations as models of dietary adaptation, examining how genetic variations and microbiota—shaped by their distinct environment-specific diets— influence metabolic health, especially when shifts to westernized diets disrupt traditional gene-diet interactions.

## 2 Genetic adaptations to diet: South Asia vs. Arctic

### 2.1 Carbohydrate metabolism

Dietary shifts have had a pivotal role in the adaptation and evolution of humans, affecting phenotypes such as lactase persistence (1, 59–61), polyunsaturated fatty acid metabolism (14, 51, 61, 62), and carbohydrate metabolism (63–65). Carbohydrates consumed today by humans are primarily derived from agriculture due to adoption of farming that triggered a rapid transition toward starch-rich diets in humans (10). However, archeological evidence suggests that plant foods containing high quantities of starch became essential for the survival of our ancestral populations already in the Pleistocene (66). Amylase genes (*AMY1* and *AMY2*) facilitate starch digestion, and higher copy number of the salivary amylase gene, also known as amylase alpha 1A (*AMY1A*), has been observed in some contemporary human populations with higher intake of starchy foods (including South Asians), compared to populations with a fishing, hunting and pastoral livelihoods, such as Arctic populations (63–65).

Plant-based diets with high starch content are broken down into simple sugars by *α*-amylase enzymes in mammals. Human genomes have three divergent, but proximal amylase genes at single locus. *AMY1A* is expressed exclusively in salivary glands, while amylase alpha 2A (*AMY2A*) and amylase alpha 2B (*AMY2B*) are expressed in the pancreas. All the three genes at amylase locus exhibits extensive copy number variation in humans (63, 67), in contrast to other great apes, who exhibit only a single copy each of the *AMY1A*, *AMY2A* and *AMY2B* genes (68), just like the Neanderthals and Denisovans (69, 70).

**Figure 3:**
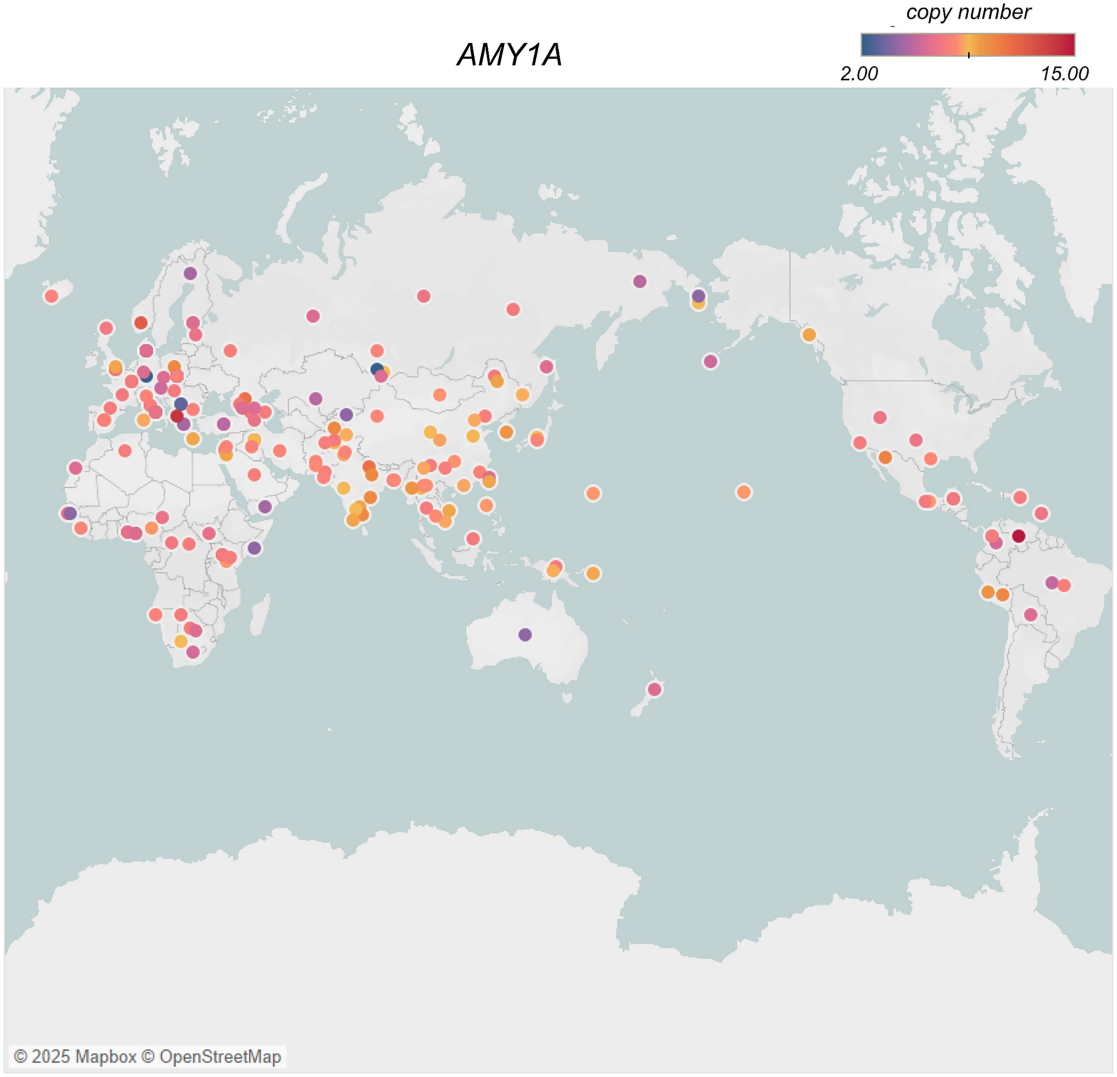
*AMY1A* gene copy numbers in different populations across the world [data from (63)] created using Tableau (https://www.tableau.com). Each circle represents a population, and the color of the circle indicates a gradient of color from lowest – 2 (blue) to highest −15 (red) average copy number of *AMY1A* gene in the population, while the yellow indicates the mid-point of copy number distribution.

Traditional diets in South Asia, rich in grains and legumes, align with genetic adaptations like the amylase gene copy number variation (*AMY1A*), which supports enhanced starch digestion (64). A recent study estimated the copy number of diploid *AMY1A* ranging from 2 to 15 across the world (Figure 3), with the highest numbers observed in Oceanic, East Asians and South Asians populations (63). *AMY1A* copy number is correlated with salivary amylase protein content in humans. Thus, the higher *AMY1A* copy numbers in populations with starch-based diets, suggest a long history of genetic selection for efficient carbohydrate metabolism. Although the expansion of *AMY1A* copy numbers has been associated with the advent of farming during Neolithic period, there has been disagreement about the timing and significance of *AMY1A* gene duplication in relation to starch-rich diets and human evolution (14, 63, 69, 71). However, a recent study resolved this locus at nucleotide-level per population, conducting evolutionary genetic analyses on ancient human and archaic hominin genomes to investigate the timing of *AMY1A* gene duplications within the context of agriculture, unraveled that the coding sequence of *AMY1A* copies are evolving under negative selection (65). The study also found a common three-copy haplotype between archaic hominins and humans, which dates back to 800 KYA, is the causal factor of rapidly evolving rearrangements through recurrent non-allelic homologous recombination. The estimated significant increase in the occurrence of haplotypes with more than three *AMY1A* copies, among European farmers over the past 4,000 years, re-affirms the potential adaptive association of *AMY1A* gene copy numbers and the starch-rich diets (65).

Conversely, Arctic populations have traditionally consumed diets rich in proteins and fats, with minimal carbohydrates due to limited plant availability in their environment. This dietary pattern of minimal-starch intake shows evidence of relaxed selection, which is reflected in lower copy numbers of *AMY1A* and *AMY2A* genes observed in Siberian populations, consistent with reduced significance of starch in the Arctic region after the dietary shifts to low carbohydrate diets (69).

Some studies have observed that a high copy number of *AMY1A* gene associates with a favorable metabolic profile, i.e., lower obesity risk and enhanced glucose absorption (72). While people with lower *AMY1A* copy number are prone to obesity and higher glucose levels (Table 1) after starchy food (63, 64, 72). Thus, an Arctic person with low *AMY1A* may experience higher glycemic stress from the same starchy meal as a South Asian with high *AMY1A*, increasing their risk of insulin resistance over time. In South Asia, the issue is reversed: urban Indian diets have shifted to refined grains and sugary drinks, which digest quickly and overwhelm even high amylase. The traditional practice of eating whole grains and high-fiber flatbreads, which slow absorption, is declining. Despite genetic predisposition, South Asians face an epidemic of diabetes due to excessive calorie and sugar intake.

**Table 1:** List of a few diet-related genes adapted distinctly to the historical food patterns of South Asia and Arctic region and their health implications.

Region	Adapted gene	Variant	Historical dietary benefit	Modern health implications	Citations
South Asia	*AMY1A*	Copy number variation	Enhanced starch digestion	Increased risk of hyperglycemia with refined carbohydrate diets	(63, 65)
South Asia	*LCT/MCM6*	*rs4988235*	Adult lactose digestion	Increased lactose intolerance and nutritional deficiencies	(78)
South Asia	*FADS2*	*rs66698963*	Enhanced omega-3 synthesis from plants	Elevated inflammation from excess omega-6 fatty acids	(20, 21)
Arctic	*FADS1*	*rs7115739; rs174570*	Efficient use of marine-derived fatty acids	Increased cardiovascular risks with shifts to non-marine fats	(51, 99)
Arctic	*TBC1D4*	*rs61736969*	Glucose regulation on low-carb diets	Significantly increased diabetes risk under high-carb diets	(109)
Arctic	*CPT1A*	*rs80356779*	Optimal ketogenesis and fat metabolism	Increased risk of hypoglycemia, particularly among infants	(105)
Arctic	*UCP1*	*rs1800592*	Enhanced thermogenesis and cold tolerance	Potential increased obesity risk with reduced exposure to cold stress and changes in diet	(116)

*AMY1A*, amylase alpha 1A gene; *LCT*, Lactase gene; *MCM6*, minichromosome maintenance complex component 6 gene; *FADS2*, Fatty acid desaturase 2 gene; *FADS1*, Fatty acid desaturase 1 gene; *TBC1D4*, *TBC1* domain family member 4 gene; *CPT1A*, carnitine palmitoyl transferase 1A gene; *UCP1*, uncoupling protein 1 gene.

### 2.2 Lactase persistence and lactose metabolism

**Figure 4:**
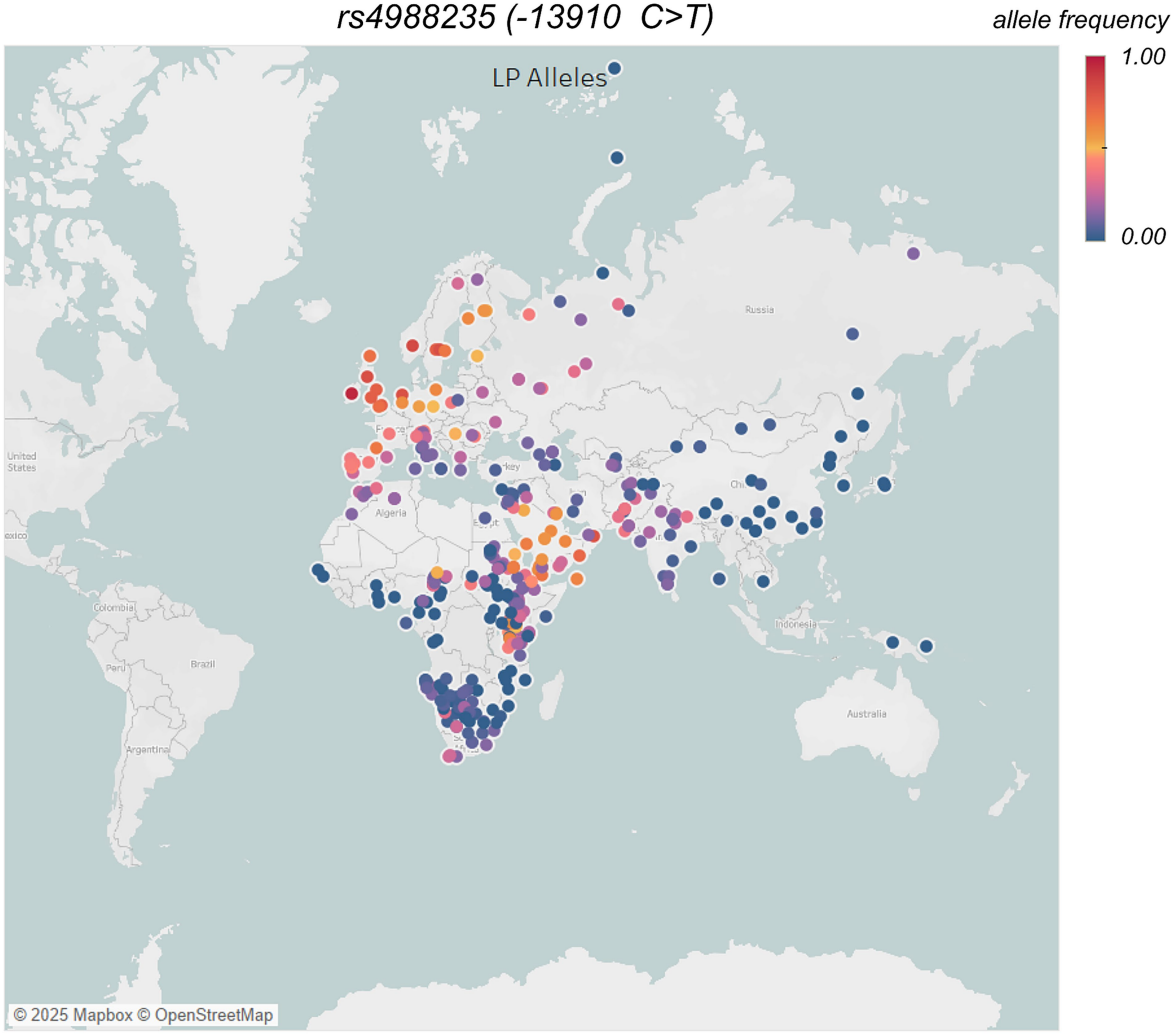
Frequency of main LCT/MCM6 allele −13,910C > T (rs4988235) in different populations across the world, data taken from UCL’s LP database – GLAD (https://www.ucl.ac.uk/biosciences/gee/molecular-and-cultural-evolution-lab/global-lactase-persistence-association-database-glad), and additional allele frequency estimation of Arctic groups from (162) data. Each circle represents a population, and the color of the circle indicates a gradient of color from low (blue) to high (red; i.e., 0–100%) occurrence of average allele frequencies. Global distribution map was generated using Tableau (https://www.tableau.com).

Lactase persistence (LP)—is a genetically inherited trait that enables humans to digest milk in adulthood by cleaving the lactose, found in mammalian milk, into absorbable glucose and galactose with the help of the lactase enzyme. Whereas incomplete digestion of milk lactose makes individuals lactose intolerant that causes gastrointestinal discomfort or diarrhea (73). Lactase non-persistence (LNP) is the ancestral state for humans, as dairy-based diets were mostly unavailable to wild mammals in adulthood and lactase production stops once individuals are weaned from mother’s milk (74–76). LP is one of the most strongly selected gene traits in recent human evolution and is observed frequently in populations with a long history of dairy consumption, making it an exemplary candidate of gene-diet co-evolution. LP is prevalent among Europeans (Figure 4) and populations with significant pastoralist ancestry, including northern South Asians (77). Interestingly, numerous human populations whose ancestors had access to milk do not have lactase persistence, such as, Central Asian herders, despite their significant consumptions of dairy products (78). The majority of human populations are lactose intolerant; nonetheless, the consequential symptoms of consuming milk or dairy products vary among individuals (79).

The rise of pastoralism (animal domestication) ~ 10 KYA in the Middle East and North Africa, which led to human consumption of dairy, resulted in strong selection pressure—enabling milk digestion beyond weaning—that likely drove the spread of LP (80). Nevertheless, the LP only reached appreciable frequencies during the Bronze and Iron Ages (59, 81–83), consistent with selection beginning in the Early Neolithic (83, 84). Consequently, the human genome shows strong signatures of selection in the region that harbors genetic variants in the minichromosome maintenance complex component 6 (*MCM6*) gene, adjacent to the regulatory regions of the lactase gene (*LCT*)—a lactase enzyme-coding locus (85, 86). While multiple genetic variants contribute to lactase persistence (LP) across different populations (77, 87), the SNP *-13,910 C > T (rs4988235)* on chromosome 2 is the most significantly associated Eurasian LP-causing allele (85, 88). It exhibits a highly structured geographic distribution in modern Europe and South Asia (89, 90). This Eurasian specific LP variant supposedly emerged in Ukraine during late Neolithic time (78) and later spread across Eurasia, likely driven by the migrations of steppe herders across and outside Europe (59, 81).

Population diversity of LP-associated SNPs and the habitation of LP populations in diverse climatic and geographical regions indicate that geography-based selection pressures individually shaped causal variants in different populations, rather than a common evolutionary adaptation. A recent study (91) found no strong link between historical milk intake and the genetic spread of lactase persistence (LP) in ancient and modern Europeans. It suggests that LP’s increased prevalence was due to rising diseases and famines associated with farming, rather than milk exploitation alone. The study also found that population fluctuations, settlement density, and wild animal exploitation are stronger drivers of lactase persistence selection than milk exploitation.

Nevertheless, South Asian populations display a heterogeneous frequencies of LP (Figure 4), and European *LCT/MCM6* allele −13910*T(rs4988235), varying from 0 to ~48% with Northwestern Indians from traditional dairy herding communities have high LP, in contrast to numerous, mostly lactose intolerant, southern/eastern non-pastoral populations (89). The dairy pastoralism in South Asia has been associated with the domestication of zebu cattle and buffalo during the Bronze age Indus Valley (~4–5 kya) (89). The earliest appearance of the −13910*T allele (*rs4988235*) in ancient Indus samples from ~2000 years before present (92) indicates a shared origin with Europe, which is mostly linked to prehistoric migrations of steppe herders.

In contrast, Arctic indigenous groups like the Inuit and Yup’ik traditionally lacked dairy animals and had little milk consumption history. Consequently, it is unsurprising that lactase persistence alleles are rare among Arctic people (Figure 4), and lactose intolerance is common in adulthood (90). Any presence of lactase persistence in Arctic communities today is likely a result of recent admixture with non-indigenous people, rather than indigenous evolution. This stark difference is often interpreted as a signature of strong selective pressure for the *LCT* variant in milk-dependent cultures. However, the link between lactase persistence and dairy reliance is not straightforward—for example, most Central Asian herders are lactase non-persistent despite a long history of dairy consumption, likely due to cultural adaptations such as milk fermentation or changes in the colonic microbiome, rather than genetic evolution (78).

Researchers estimate that lactase persistence conferred a 5–10% per-generation fitness advantage in pastoral populations. Conversely, populations with low lactase persistence allele frequencies are more likely to experience an increase in lactose intolerance symptoms, such as bloating, diarrhea, and gas (90). In traditional South Asian culture, dairy is a staple food, but not everyone is lactase persistent. Even those without the *LCT* mutation can consume lactose through food processing, such as fermenting milk into yogurt or sour milk (lassi), which reduces its lactose content. If urbanization causes people with low levels of the LP allele to consume more unfermented milk and dairy products (like store-bought pasteurized cow’s milk, ice cream, cheese), we can expect an increase in symptoms of lactose intolerance, such as bloating, diarrhea, and gas. Lactose intolerance itself is not life-threatening, but it can discourage dairy consumption in populations already at risk of calcium and vitamin D deficiency, particularly those in the Arctic, where limited sunlight and alternative calcium sources make it difficult to meet nutritional needs.

### 2.3 High-fat (lipid) metabolism

The diet of Arctic populations has traditionally consisted of animal-based foods, including reindeer, fish, and marine mammals. The reliance on fat as a primary energy source in Arctic populations, living in environments with limited access to carbohydrates, resembles the macronutrient profile of a ketogenic diet, though physiological ketosis may be modulated by other dietary and genetic factors, unlike a “ketogenic diet,” which implies a deliberate dietary regime to induce ketosis. Arctic populations exhibit adaptations that allow them to extract the maximum energy from fat, ensuring survival through long periods of food scarcity (51).

The fatty acid desaturase (*FADS*) gene cluster, comprising *FADS1*, *FADS2*, and *FADS3*, encodes enzymes called fatty acid desaturases—vital for converting dietary fatty acids into forms that the body can utilize. Particularly, *FADS1* encodes Δ5-desaturase, while *FADS2* encodes Δ6-desaturase (93). Together, these enzymes enable the synthesis of long-chain polyunsaturated fatty acids (LC-PUFAs) from plant-derived short-chain (SC-PUFAs) precursors, such as linoleic acid (LA) and *α*-linolenic acid (ALA) (94). These LC-PUFAs include omega-6 fatty acids like arachidonic acid (AA), omega-3 fatty acids like EPA and DHA. Long-chain polyunsaturated fatty acids (LC-PUFAs) are crucial for cell membranes, particularly in the brain and retina, and for producing eicosanoid signaling molecules (95). Humans must get LC-PUFAs from their diet (e.g., from animal foods) or make them inside their bodies through the FADS pathway. Diets rich in seafood and meat provide ample LC-PUFAs, potentially reducing reliance on endogenous synthesis and thus may require lower levels of *FADS1* and *FADS2* activity. Conversely, plant-rich diets such as oils, nuts, seeds providing only SC-PUFAs, hence, demanding a more efficient conversion by FADS enzymes to avoid deficiencies (20, 21, 51, 62, 96, 97). This difference in environmental interaction set the stage for regional adaptation in *FADS* genes.

The *FADS1* and *FADS2* genes are located close to each other on chromosome 11. Of the two LD blocks at this locus, LD block 1 comprises two most studied haplotypes—the ancestral (A) and derived (D) haplotypes (96). Haplotype D likely increases *FADS1* expression and is thus advantageous for plant-based diets, whereas haplotype A is linked with animal-based diets (96). Variations in these haplotypes result in differences in the efficiency of PUFA synthesis, which has significant implications for dietary adaptation.

While haplotype D is common in present-day Africans (62, 96), its increase in frequency in European and South Asian populations appears linked to the rise of agriculture and plant-based diets during the Neolithic (59). As diets shifted to rely more heavily on plant-based foods requiring enzymatic conversion of SC- to LC-PUFA, the selection for haplotype D increased. Haplotype A is predominantly found among indigenous populations of the Arctic, Siberia, and Native Americans (98, 99). Paleogenetic evidence suggests that the widespread occurrence of haplotype A began with the migration of Upper Paleolithic humans into Eurasia. These populations subsisted on diets rich in animal lipids, reducing the selective pressure for PUFA synthesis (100).

**Figure 5:**
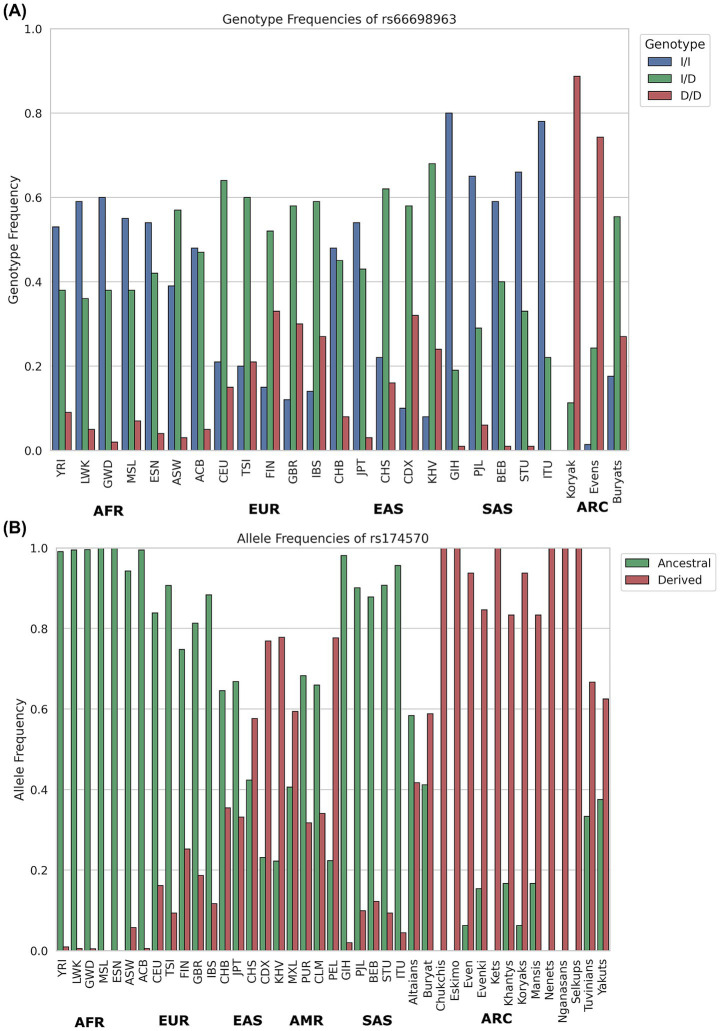
Global distribution of the so-called ‘vegetarian diet variant’ *rs66698963* — frequent in South Asia, and the ‘marine diet variant’ *rs174570*—with the highest derived allele frequency in Arctic populations. **(A)** Genotype frequency distribution bar plot of *rs66698963*—22 bp indel polymorphism, insertion/insertion (I/I), insertion/deletion (I/D), deletion/deletion (D/D) based on the data retrieved from Kothapalli et al. (20) and Malyarchuk et al. (163). **(B)** Allele frequency distribution of rs174570 in global populations, including data from the 1000 Genomes Project populations retrieved from (21) and Arctic groups from (162). AFR-Africans, EUR-Europeans, EAS-East Asians, AMR-Americans, SAS-South Asians, ARC-Arctic regions.

In Arctic populations, the prevalence of haplotype A is exceptionally high. For instance, it has reached near fixation among the Greenland Inuit (51). This adaptation reflects the lipid-rich traditional diets of these groups, where dietary PUFAs from marine and terrestrial animals were abundant, eliminating the need for elevated enzymatic activity in PUFA biosynthesis (51, 96, 100). The distribution of haplotypes A and D reflects dietary adaptations shaped by environmental pressures. The high prevalence of haplotype A in Arctic populations underscores its role in supporting traditional lipid-rich diets, while the emergence and dissemination of haplotype D in agricultural societies, such as South Asia, highlight its importance for plant-based dietary adaptations. Among the numerous mutations present on LD block 1 across these haplotypes, a few outliers have emerged as the strongest candidates for positive selection, both in South Asia and Arctic populations. South Asians display strong evidence of positive selection for an indel, *rs66698963—*a 22 bp insertion in the *FADS2* gene regulatory region—highly frequent in South Asians (~70%) and in some African (53%) and East Asian (29%) populations (Figure 5) with a long history of vegetarian diets (20, 21). This insertion allele is believed to have enabled South Asians to synthesize LC-PUFAs more efficiently by enhancing the expression of *FADS2*/*FADS1*, potentially as an adaptation to a more vegetarian diet. Interestingly, this insertion was found to be deleted in the Greenlandic Inuit populations.

In Greenlandic Inuit, two most differentiated SNPs, *rs7115739* and *rs174570*, both located in *FADS2* (toward the *FADS1* gene region), were linked to decreased LC-PUFAs and increased SC-PUFAs (51). Inuit traditionally consume extremely high levels of LC-PUFAs from fish and marine mammals, which ostensibly reduces their need to synthesize these fatty acids endogenously. However, they have a low intake of some SC-PUFAs, such as LA. Interestingly, the derived alleles of these variants that have ~100% frequency in Arctic Inuits (Figure 5) but very low in other populations, appear to compensate for this deficiency by down-regulating the omega-3/omega-6 fatty acids synthesis pathway (51). Interestingly, South Asia has prevalent frequency of the ancestral allele of the functionally most significantly associated SNP—*rs174570*. Interestingly, one of the Arctic Inuit-selected SNPs *(rs174570)* is rare elsewhere, but the ancestral version of it was also favored in South and East Asians (in strong LD with the insertion allele—*rs66698963*). This suggests a case of parallel adaptation to dietary extremes through distinct, population-specific genetic changes: the ancestral allele, combined with the insertion, helps vegetarians better metabolize their diet, while the derived allele is best suited for marine diets (20).

The *FADS2* insertion allele in South Asians is believed to be an adaptive response to plant-based diets that provides a metabolic advantage by ensuring sufficient AA and DHA synthesis from vegetarian food sources (20, 21). On the contrary, the Inuit exemplify a population where relaxation of selection (or even reverse selection) for endogenous synthesis occurred. The derived *FADS* alleles common in Inuit reduce levels of certain unsaturated fatty acids, which was likely beneficial in their ecology, where they consumed large amounts of seal, blubber, and fish oil daily. The evolution of *FADS* is a story of balancing metabolic needs with available foods. For instance, a vegan diet requires a certain ability to produce LC-PUFAs, while a carnivorous diet may be wasteful or harmful if there is an overproduction. Ancient DNA (aDNA) supports these timelines: selection tests on aDNA show that variants in *FADS* were selected before and after the agricultural revolution in Eurasia (59). In summary, *FADS* adaptations demonstrate how human populations adapted their lipid metabolism to suit their local diet composition.

The prevalent South Asian insertion allele, near *FADS2*, might cause chronic inflammatory diseases in the vegan/vegetarian diet due to high precursor (LA) consumption (20). People with this genotype, common in South Asia, may convert a lot of omega-6 (LA) into large amounts of AA. Having too much AA compared to omega-3 can cause inflammation, which may increase the risk of cardiovascular disease. This may partly explain why South Asians in urban areas have high rates of heart disease at a young age. They often consume oils high in omega-6 (e.g., soybean, sunflower oil) while their intake of omega-3 is low. Their efficient *FADS* alleles convert that omega-6 into pro-inflammatory eicosanoids. India’s coronary artery disease and diabetes rates are rising sharply as traditional diets with balanced fatty acids from mustard oil and ghee are replaced by trans fats and cheap seed oils.

Inuit with adaptive alleles near *FADS1* have lower LDL cholesterol and shorter height, indicating a broad impact on metabolism. These variants likely helped Inuit avoid blood lipid overshoot and maintain cardiovascular health despite their high-fat diet (51). Inuit, despite their high fat intake, have traditionally had low rates of ischemic heart disease and type 2 diabetes, which puzzled researchers until genetic factors were identified.

#### 2.3.1 Ketogenesis, energy utilization and genetic adaptation

Ketogenesis is the process by which fatty acids are converted into ketone bodies, providing an alternative energy source when glucose is unavailable due to food scarcity. This metabolic pathway is essential for cold-adapted populations, who often face long periods of low carbohydrate intake during the winter (101). Ketone bodies, such as beta-hydroxybutyrate (BHB), serve as a stable energy source for the brain and muscles, allowing individuals to maintain cognitive and physical performance during periods of fasting (102). In addition to providing energy, ketones have neuroprotective properties, helping to prevent cognitive decline during prolonged periods of caloric deprivation. The ability to switch between glucose and fat metabolism is a key feature of metabolic flexibility in cold-adapted populations (103). Such an episodic food scarcity has likely driven the adaptation in genes such as carnitine palmitoyl transferase 1A (*CPT1A*) and TBC1 domain family member 4 (*TBC1D4*) in Arctic populations.

Carnitine palmitoyltransferase 1A (*CPT1A*) gene encodes for an enzyme that is essential for converting long-chain fatty acids into energy, especially during fasting. In Arctic populations relying on a high fat diet, fatty acid metabolism and ketogenesis are crucial for survival during prolonged food scarcity and extreme cold. The strong signals of positive selection of a nonsynonymous variant (*rs80356779*) in *CPT1A* observed in Arctic populations of Northeast Siberia, North America and Greenland, suggest this variant, also dubbed as the ‘Arctic variant’ (104), has been a target of adaptation to extreme cold and episodic food scarcity (105). The derived allele of this gene has reached high frequencies in circum-Arctic populations (including 68% in Northeast Siberians). Paradoxically, the same variant, when present in the homozygous state, has been described in medical genetic literature as deleterious for infants on a modern carbohydrate-rich diet, as reflected in its association with hypoketotic hypoglycemia (106) and high infant mortality (104). Indigenous populations of Northeast Siberia, Alaska, Canada, and Greenland show significantly higher infant mortality than non-indigenous populations of the same regions (107). These lines of evidence suggest that a powerful selective sweep (estimated at 6–23 ka) driven by the advantages this variant may have conferred in response to a high-fat diet or cold environment had to overcome its fitness reducing effects, highlighting a striking example of recent human adaptation with potential trade-offs. *CPT1A*, the rate-limiting enzyme in fatty acid oxidation (FAO) that provides an alternative energy source for the survival of cancer cells, plays a crucial role in metabolic adaptation in cancer pathogenesis (108).

The *TBC1D4* gene mutation (*rs61736969*), predominantly found in Arctic populations (~17% in Inuit), facilitates glucose regulation under historically low-carbohydrate diets (109). The Greenlandic Inuit-specific *TBC1D4* loss-of-function variant (*TBC1D4* c.2050C > T) that impairs *GLUT4* glucose uptake in muscle after carbohydrate intake, likely rose in frequency because under low-carb traditional diets it had a favorable effect on fasting glucose availability. However, this variant significantly elevates blood glucose levels and diabetes risk in its Arctic carriers who eat carbohydrate-rich meals (109, 110). This variant has almost negligible occurrence in South Asians, East Asians and Europeans but is somewhat present in Americans (111).

#### 2.3.2 Cold-induced thermogenesis, brown adipose tissue and ethnic variation in metabolic adaptation

Cold-induced thermogenesis, the process by which the body generates heat in response to cold exposure, is primarily mediated by brown adipose tissue (BAT) and uncoupling protein 1 (*UCP1*) (112). BAT facilitates non-shivering thermogenesis, enabling the generation of heat without significant calorie expenditure, which is crucial for maintaining core body temperature in cold environments. In Arctic populations, such as the Siberians, BAT plays a vital role in sustaining body temperature during extended cold exposure, allowing survival in sub-zero conditions with limited food intake (112).

Recent findings suggest that BAT activity contributes to the metabolic adaptations observed in populations regularly exposed to cold environments (113). In contrast, populations living in South Asia, Africa, and other regions where extreme cold exposure has been largely absent have reduced BAT activity and lower basal metabolic rates (114). The *UCP1* gene polymorphisms at −3,826 position (*rs1800592*) involved in brown adipose tissue (BAT) regulation (115) has been linked to obesity in populations lacking a historical exposure to cold environments (116). A slower metabolism is associated with reduced activity in non-shivering thermogenesis (NST) and brown adipose tissue (BAT) (117). People of African ancestry have been associated with lower basal metabolic rates, predisposing them to higher risks of obesity and hindering of weight loss (118, 119). Interestingly, reduced basal metabolic rates have been documented in young African American women of normal weight and without prior weight issues, indicating a potential predisposition to obesity (120).

South Asian ancestry has also been associated with reduced BAT volume and NST and, hence, a decreased basal metabolism and energy expenditure (121). Like Africans, South Asians living in Western countries are more prone to obesity and metabolic disorders such as diabetes and cardiovascular diseases, which, besides socio-economic and cultural differences, may be linked with reduced BAT activity. It is likely that the lack of ancestral exposure to cold is the main reason for the reduced BAT activity in these ethnic groups.

Contrarily, East Asian ancestry (e.g., Chinese, Japanese, and Korean) has been linked by some studies with relative obesity resistance (122–124). Despite adopting a Western lifestyle, Chinese people living in industrialized countries have been found to have some of the lowest rates of obesity. In the US, East Asians are the least likely to be obese among all ethnic groups, probably owing to the ancestors of East Asia who lived under cold exposure for thousands of years and adapted to extreme cold (125).

The modern obesity pandemic is highly complex, with multiple puzzling questions. However, it’s clear that obesity rates vary greatly by ethnicity, and this is reflected in basal metabolic rates which suggest that the daily caloric requirements for each ethnicity vary. Thus, necessitating an ethnicity-specific guidelines on caloric requirements to prevent overeating, and obesity related diseases. Indeed, a similar target approach of ethnicity-specific guidelines for clinical cardiovascular measures like HDL and LDL cholesterol has been issued for South Asians, based in US, in recent years (126).

## 3 Environmental pressures and diet-microbiome interactions

The human intestine harbors a complex and diverse microbial ecosystem of bacteria, archaea, viruses, and other microorganisms, collectively referred to as the gut microbiota (127). To date, over 5,000 microbial species have been identified in the human gut globally, with individuals’ microbiomes typically comprising approximately 150 to 400 bacterial species (128). This gut microbiota plays a crucial role in our health by forming a protective barrier against pathogens, producing bioactive metabolites, and helping to modulate our immunological responses (129, 130). Gut microbiota dysbiosis has been linked with several diseases including inflammatory bowel diseases, allergies, obesity, autoimmune diseases (131), cancer (132), cardiovascular diseases (133), and diabetes (134). However, in general, the homeostasis of the gut ecosystem is upheld by core bacterial species commonly shared among different individuals (135, 136). Although a certain level of stability emerges with the establishment of the gut microbiota and its diversity in healthy adults, the microbiome remains a dynamic system throughout life, continuously shaped by diet, medication use, lifestyle and other environmental factors (137–139).

Recent studies have shown that geographic location is one of the main factors shaping the composition and diversity of the human gut microbiota, exceeding the influence of diet and lifestyle (164–166). Diet plays a significant role in shaping the gut microbiome, with changes occurring even within 24 hours of consuming a different diet (140). Prolonged dietary changes can lead to long-term and permanent changes in the gut microbiome (141). South Asian microbiomes have been found to be enriched with bacteria that digest fibers and plant polysaccharides, while Arctic microbiomes are optimized for animal fat digestion (142). These microbiome adaptations play a crucial role in nutrient absorption and energy balance, reflecting the influence of diet and environment on microbial composition.

The human gut microbiota can be clustered into distinct enterotypes, independent of nationality, age, or gender. These enterotypes are most notably characterized by the dominance of genera such as *Prevotella* or *Bacteroidetes*, which have been associated with long-term dietary patterns—Prevotella with plant-rich, high fiber diets and Bacteroides with diets high in animal protein and fat (143, 144). Vegetarian diets often with dairy (lacto-vegetarianism), traditionally consumed by most South Asians, especially Hindu and Jain followers, are richer in fiber and lower in saturated fat and protein compared to high-fat animal diets. This diet, rich in fiber and polyphenols, is believed to support a more diverse ecosystem of beneficial bacteria (145). It increases the levels of bacteria that can break down complex carbohydrates and polysaccharides into SCFAs, which are essential for regulating metabolism, inflammation, and disease (146). Consequently, vegetarian South Asians have experienced an increase in *Prevotella, Roseburia, Lactobacillus*, and *Bifidobacterium,* which are associated with the fermentation of dietary fiber and the production of SCFAs (145, 147).

In contrast, the diets of non-vegetarian South Asians, who consume animal proteins like poultry, fish, and goat alongside heavy plant-based foods typical of South Asian cuisine (148), include higher saturated fats and proteins than vegetarians while remaining relatively more plant-heavy compared to Western omnivorous diets. Southern Indian non-vegetarians have a higher ratio of *Bacillota* (formerly *Firmicutes*) and *Bacteroidota (formerly Bacteroidetes)* compared to vegetarians, characterized by a higher abundance of *Bacteroides, Ruminococcus,* and *Faecalibacterium* (148). *Bacteroides* is commonly linked to protein and fat metabolism and is frequently enriched in gut microbiota profiles of individuals in the Western diet (149). They also have lower microbial diversity compared to vegetarians, due to reduced plant variety, but still higher than that of individuals consuming Arctic diets (150).

Traditional diets of Arctic populations—rich in animal-based foods often consumed raw, cooked, dried, frozen, or fermented—contribute to a unique gut microbiome composition that supports their nutritional and health needs in cold environments (151). Arctic Inuit have enriched *Clostridium*, *Bilophila*, and *Enterobacteriaceae*, which metabolize animal fats and proteins but a low level of *Prevotella,* and *Faecalibacterium* which is fiber-dependent and anti-inflammatory (150, 152). Microbiome community structure and diversity of the Inuit have been found to be similar to Western Eurasians, with no significant difference in the relative abundance of Clostridia and Bacteroides (152). However, subtle but significant differences were observed in the relative abundances and diversity of certain microbial taxa and strains, particularly *Prevotella* and *Akkermansia,* which appear to be influenced by diet and geography. High-fat diets are associated with low *Prevotella* species diversity along with deficiency in *Bifidobacterium*, *Lactobacillus*, and *Akkermansia* (153). However, traditional Arctic diets rich in omega-3 fatty acids have been shown to promote the growth of beneficial bacteria involved in anti-inflammatory pathways such as *Faecalibacterium* (154) and *Roseburia* (155), essential for reducing inflammatory bowel disease (IBD) and enhancing cardiovascular health.

Nevertheless, the ongoing shift toward westernized diets enriched with refined carbohydrates and processed foods, significantly disrupts gut microbiome adaptations, increasing susceptibility to chronic diseases (152, 156). The loss of beneficial bacterial species and the consequent decrease in short-chain fatty acid (SCFA) production have been associated with impaired gut barrier function and increased inflammation, which in turn elevate the risk of obesity, cardiovascular diseases and cancers (157, 158). Similarly, the high trimethylamine N-oxide (TMAO), associated with animal-protein-rich diet and metabolized by *Bacteroides* and *Clostridium*, is linked to atherosclerosis, chronic kidney disease and metabolic diseases (55, 133, 159). The production of higher levels of secondary bile during saturated fat and bile acid metabolism, mediated by *Bilophila* and *Clostridium*, may increase the risk of colon cancer (149). Thus, preserving traditional diets, or integrating elements thereof, could help maintain a gut microbiome that supports health in indigenous populations.

Despite significant strides in understanding the impact of gut microbiota on metabolic health, our insights remain fragmented. While previous studies have established associations between the microbiome and key metabolic factors, such as obesity and glucose regulation (156), a thorough comprehension of the functional mechanisms by which the microbiome influences these processes, considering the local factors (diet, environment), is still required.

## 4 Implications for chronic disease risk

The widespread adoption of Western diets rich in processed foods, sugars, and saturated fats has greatly disrupted traditional gene-diet-microbiome interactions that historically optimized health in populations like those of South Asia and the Arctic (Table 1). The disruption has had profound consequences, as evident in the stark regional disease burdens reported by WHO in 2021 (Table 2). This highlights the critical mismatch between genetic and microbial adaptations and contemporary dietary practices.

**Table 2:** Leading causes of death (≥1% of total deaths) for South and Southeast Asian and European (includes Arctic) region as per the WHO report 2021.

WHO South-East Asia Region 2021			WHO European Region 2021		
Rank	Cause	% of total deaths	Rank	Cause	% of total deaths
0	**All Causes**	**100,0**	0	**All Causes**	**100,0**
1	COVID-19	21,4	1	Ischaemic heart disease	18,9
2	Ischaemic heart disease	11,4	2	COVID-19	15,5
3	Stroke	8,2	3	Stroke	9,2
4	Chronic obstructive pulmonary disease	6,8	4	Alzheimer disease and other dementias	4,3
5	Tuberculosis	3,4	5	Trachea, bronchus, lung cancers	3,7
6	Diarrheal diseases	3,2	6	Chronic obstructive pulmonary disease	2,8
7	Lower respiratory infections	2,8	7	Colon and rectum cancers	2,3
8	Diabetes mellitus	2,6	8	Hypertensive heart disease	2,1
9	Cirrhosis of the liver	2,3	9	Lower respiratory infections	2,1
10	Kidney diseases	1,5	10	Diabetes mellitus	1,8
11	Preterm birth complications	1,4	11	Cirrhosis of the liver	1,6
12	Asthma	1,4	12	Kidney diseases	1,5
13	Hypertensive heart disease	1,4	13	Breast cancer	1,5
14	Alzheimer disease and other dementias	1,1	14	Cardiomyopathy, myocarditis, endocarditis	1,3
15	Rheumatic heart disease	1,1	15	Pancreas cancer	1,3
16	Mouth and oropharynx cancers	1,0	16	Prostate cancer	1,1
17	Trachea, bronchus, lung cancers	1,0	17	Stomach cancer	1,1

Web sources. GLAD, https://www.ucl.ac.uk/biosciences/gee/molecular-and-cultural-evolution-lab/global-lactase-persistence-association-database-glad. WHO health report 2021, https://data.who.int/countries.

### 4.1 South Asia

Historically, the traditional South Asian diet, rich in complex carbohydrates, dietary fiber, and plant-derived nutrients, facilitated adaptations such as increased *AMY1A* gene copy numbers and efficient fatty acid metabolism (Table 1). Our modern diet, characterized by the shift toward calorie-dense, highly processed foods, exacerbates genetic predispositions to metabolic disturbances, particularly insulin resistance and impaired carbohydrate metabolism (126, 160). These disruptions have been directly linked to the rapid rise in rates of type 2 diabetes, obesity, and cardiovascular diseases, which are now among the leading causes of mortality in the WHO South-East Asian region (Table 2). Moreover, while high carbohydrate metabolism efficiency was once advantageous, it may now predispose individuals to hyperglycemia when exposed to refined sugars, prevalent in modern diets, further aggravating diabetes risk.

### 4.2 Arctic regions

Arctic populations traditionally thrived on nutrient-dense, high-fat diets rich in marine mammals and fish. These diets facilitated genetic and microbiome adaptations crucial for efficient lipid metabolism and glucose regulation. (e.g., *FADS* haplotype A, *CPT1A*, *TBC1D4* variants; Table 1). However, recent dietary transitions toward Western diets, characterized by higher refined carbohydrates and lower marine-derived nutrients, pose significant metabolic health threats. These dietary shifts have been strongly associated with increased prevalence of obesity, diabetes, and cardiovascular diseases (161). This is reflected in the high mortality attributed to ischemic heart disease, stroke, and related chronic conditions in the WHO European and Arctic regions (Table 2). Climate change exacerbates this disconnect by disrupting the environment and reducing access to traditional food sources. These health impacts are further amplified by environmental disruptions.

In light of these trends, a strategic preservation or reintegration of traditional dietary practices that align with regional genetic and microbiome adaptations could substantially diminish chronic disease risks. These tailored nutritional strategies would not only alleviate immediate health burdens but also fortify long-term population health resilience by harmonizing contemporary dietary environments with evolutionary genetic backgrounds.

## 5 Genome-informed precision nutrition strategies for health

Genome-based nutrition strategies have the potential to tailor dietary recommendations to an individual’s genetic profile, considering their ancestral dietary history and current lifestyle. By integrating genome and microbiome data, these strategies hold transformative potential for precision nutrition. By identifying genetic variations and microbiome features that influence nutrient metabolism and disease risk, personalized dietary interventions can be developed to promote optimal health and prevent chronic disease. These interventions could customize dietary recommendations based on genetic variants related to carbohydrate and fat metabolism (e.g., *AMY1A*, *FADS2*, and *TBC1D4*) and emphasize dietary components traditionally aligned with cultural and genetic backgrounds.

Specifically, by drawing upon regional adaptations of South Asian and Arctic populations to their vastly divergent environments (including dietary patterns), precision nutrition can generate personalized recommendations that align cultural diets with health requirements. This demonstrates the potential of region-specific genomic nutrition to mitigate chronic disease risks globally.

Consequently, we propose a comprehensive multi-omics (genomics, microbiome, and metabolomics) research approach that integrates historical dietary patterns, taking into account the traditional and religious dietary patterns, to enhance precision nutrition protocols.

## 6 Conclusion and future perspectives

In conclusion, exploring the genetic adaptations of populations in contrasting climates, like South Asia and the Arctic, underscores the importance of considering genetic diversity, local dietary traditions, religious practices, and environmental context in developing nutrition strategies.

The comparative analysis of dietary adaptations in South Asia and the Arctic highlights the remarkable variability in human nutritional needs, which are shaped by distinct evolutionary histories, and traditional and religious practices. These comparisons emphasize that nutrition strategies need to be climate-sensitive, population- and culture-specific. The rise in metabolic disorders among Arctic and South Asian communities is linked to dietary changes. Reintroducing traditional foods, promoting seasonal eating, and prioritizing nutrient-rich foods can, where economically effective and viable, reduce health risks caused by the transition to Western diets. By understanding the connection between genes, microbiome, diet, and environment, especially comparing the metabolomic impact of the traditional diets used by indigenous communities vs. westernized diet of migrants thereof, we can promote precision health and improve the well-being of people globally as an equitable approach to public health in diverse environments.

Combining genomic, microbiome, metabolomic, and archaeological (informing traditional, religious and diet practices in ancient times) data will greatly improve personalized nutrition.
